# PD34 - Childhood mastocytosis: serum baseline total tryptase levels and extent of cutaneous disease as predictors of mast cell mediator release symptoms

**DOI:** 10.1186/2045-7022-4-S1-P34

**Published:** 2014-02-28

**Authors:** Alexia Chatzipetrou, Christoforos Koulias, Christina Georgia Zeliou, Myrto Potika, Katerina Chliva, Michael Makris

**Affiliations:** 1 2nd Department of Dermatology and Venereology, Attikon University Hospital, Athens, Greece

## Introduction

Pediatric mastocytosis has generally a benign prognosis. However some patients suffer from severe mast cell (MC) mediator-associated symptoms including anaphylaxis.

## Aim

The aim of this study was to identify predictors for MC mediator release symptoms in children with mastocytosis.

## Methods

A total of 25 children (median age of onset 14,2 months (range, 0 mth–12ys) diagnosed with mastocytosis have been studied. Patients were classified according to the type of skin lesions following the WHO classification. Mastocytosis-related symptoms and data on specific triggering factors were systematically recorded for 3 years after diagnosis. Severity was graded according to standard recommendations. Serum baseline total tryptase (sbT) levels (CAP;Phadia Diagnostics) were measured at the time of referral. The extent of cutaneous disease was determined using the technique used to assess burns and is given as percentage of total body surface area (BSA).

## Results

The different subtypes of mastocytosis observed included MPCM in 17/25(68%), MC in 4/25(16%), DCM in 1/25(4%), and ISM in 3/25(12%) cases. Skin rubbing and hot bath were the most common triggers for MC-related symptoms. Other clinically relevant triggers were irritability and fever, whereas drugs, food, perioperative and vaccination were rarely involved. The most frequent symptoms were flushing (68%), diarrhea (24%), and anaphylactic reactions (36%). Four children (1 DCM, 2 ISM, 1 MPCM) had grade 4 symptoms. The median sbT for the whole group was 6 ng/dl (range, 2–378ng/dl). A significantly higher (p<0.029) median sbT was found in cases with systemic symptoms vs those without (mean rank 14.97 vs 7.93ng/dl; range, 4**–**378 vs 2**–**9.1ng/dl) and especially among patients with grade 4 symptoms (p<0.07). BSA was significantly higher (p<0.019) in patients with grade 4 symptoms. In line with these findings, the correlation of BSA to sbT levels was significant (p<0.046).

## Conclusions

Extensive cutaneous disease and higher sbT levels at disease onset can identify children at risk for severe MC mediator release symptoms. Consequently, early antimediator therapy should probably be given to these children.

